# Career perspective: Victor A. Convertino

**DOI:** 10.1186/s13728-015-0040-y

**Published:** 2015-11-19

**Authors:** Victor A. Convertino

**Affiliations:** U. S. Army Institute of Surgical Research, 3698 Chambers Pass, Building 3611, JBSA Fort Sam Houston, TX 78234-6315 USA

**Keywords:** Exercise, Heat acclimation, Space, Bed rest, Lower body negative pressure, Blood volume, Hemorrhage, Compensatory reserve, Intrathoracic pressure regulation, Medical monitoring

## Abstract

This review focuses on a career of unique opportunities to participate in various areas of research related to extreme physiology and medicine. My experience as a volunteer subject in exercise experiments conducted at NASA included the study of acute and chronic physiological responses and adaptations to exercise in environments of hypoxia, heat stress, and simulated microgravity (bed rest), and eventually to my doctoral work on mechanisms underlying expansion of plasma and blood volume with acute and repeated exercise and heat exposure. My career has taken me to research positions at NASA, the Stanford University School of Medicine, the University of Arizona, and the U.S. Air Force Research Laboratory before assuming my present location at the U.S. Army Institute of Surgical Research. As a result of these multiple experiences across a period of 45 years, I have had opportunities to translate basic research to astronauts, high-performance aircraft pilots, and critically ill patients who are challenged by conditions of extreme physiology and medicine.

## NASA, Ames Research Center (1970–1982)

**Fig. 1:**
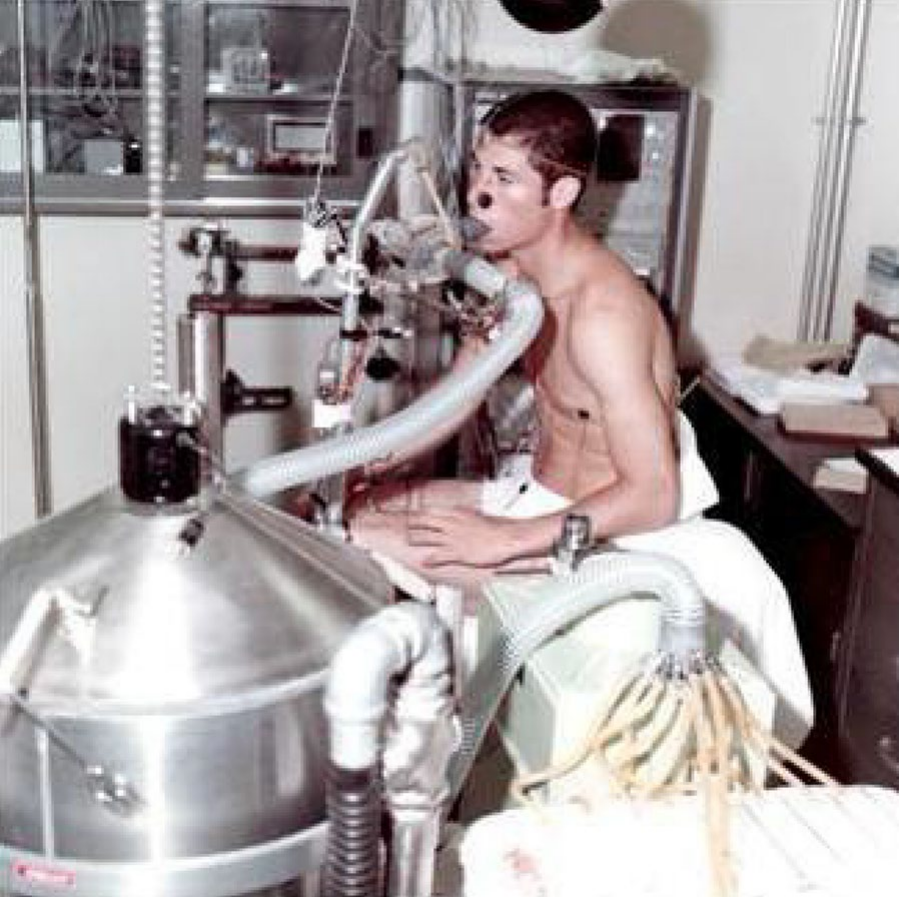
Expired air collection from subject Convertino into a tissot tank during metabolic experiments conducted in the summer of 1970 at the Laboratory of Human Environmental Physiology at NASA’s Ames Research Center

**Fig. 2:**
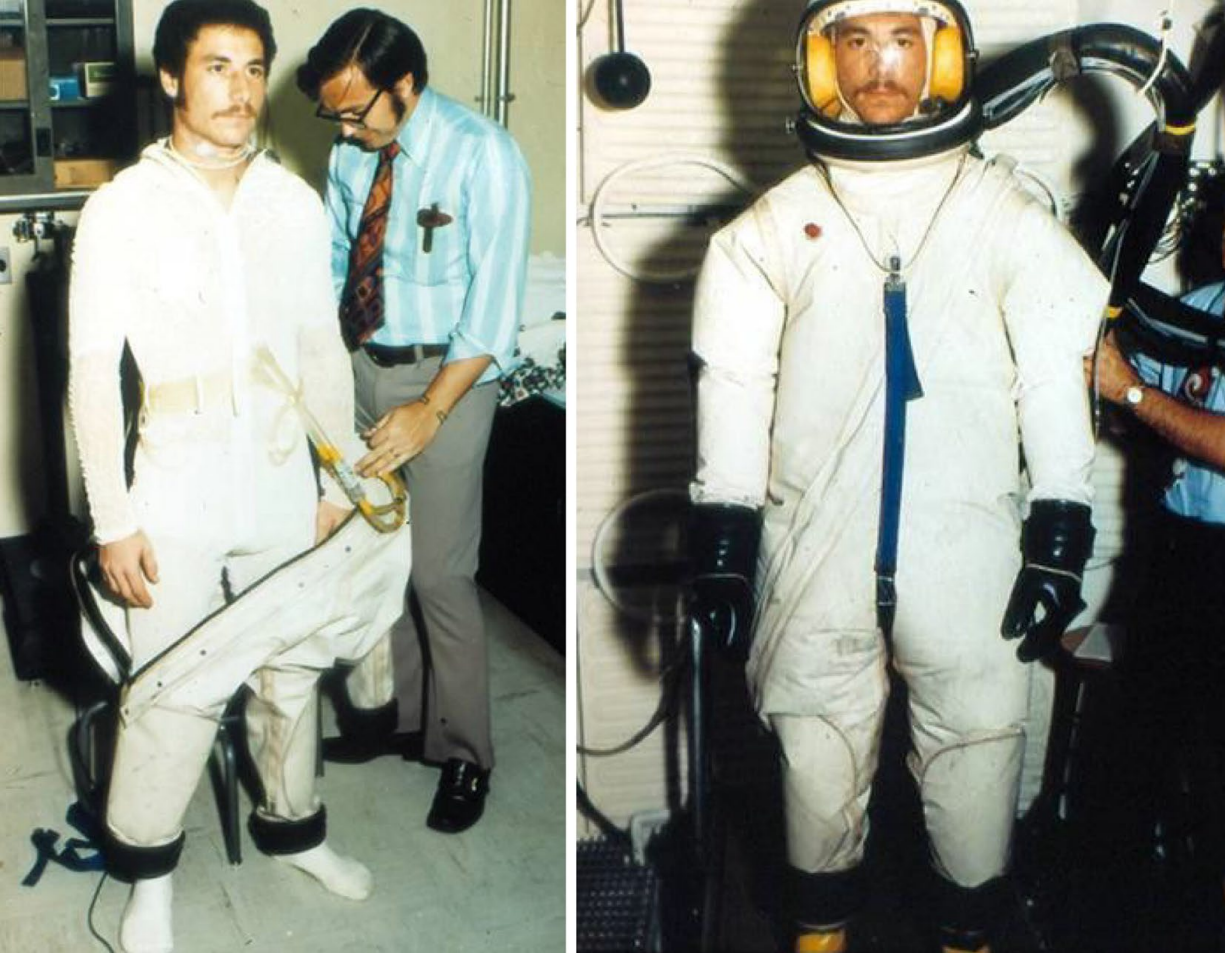
Human volunteer Convertino donning an early prototype for testing of a liquid cooling garment (LCG) developed for use by astronauts for thermoregulation during extravehicular activities in the summer of 1978 at NASA’s Ames Research Center. The late Dr. Alan Chambers, former Chief of the Man-Vehicle Systems Research Division and Director of Space Research, is instrumenting Convertino

My undergraduate and graduate studies in systemic integrative exercise and environmental physiology prepared me well for the unique opportunities to participate in various human experiments in the area of extreme physiology and medicine. My initial research experience began in 1970 in the role of a volunteer subject in exercise experiments (Fig. 1) conducted in the Laboratory of Human Environmental Physiology at NASA’s Ames Research Center under the directorship of Dr. John Greenleaf in collaboration with my undergraduate advisor at San Jose State University, Dr. James Bosco. I also had the opportunity to participate as a volunteer subject in studies to test early NASA prototypes of liquid-cooling garments that were developed as a countermeasure to avoid the serious operational issue of hyperthermia associated with extra-vehicular activity (EVA) space walks (Fig. 2). This experience became the backbone of my motivation to study human physiology in extreme conditions and led to my participation as a volunteer assistant lab staff in experiments designed to describe the acute and chronic physiological responses and adaptations to exercise in environments of hypoxia, heat stress, and bed rest as a simulation of microgravity. My subsequent enrollment to graduate school to pursue my doctoral degree in physiology at the University of California at Davis under the mentorship of Dr. Edmund Bernauer provided an opportunity to continue human environmental research in Dr. Greenleaf’s lab. My subsequent research on the study of mechanisms underlying the expansion of plasma and blood volume with acute and repeated exercise and heat exposure formed the basis for my doctoral dissertation. My dissertation research demonstrated that both physical exercise and body heat contributed separately and additively to optimal expansion of plasma volume [1].

After completing my doctoral degree, I accepted a position as a Research Associate in the Cardiology Division at the Stanford University School of Medicine. In this position, I was able to work under the mentorship of Dr. Harold Sandler, chief of the Biomedical Research Division at NASA-Ames on the study of physiological adaptation to varying gravity environments, with development of exercise training and countermeasures for astronauts. The collaboration with Dr. Norman Shumway who performed the first cardiac and heart–lung transplants at Stanford University led to the novel water-immersion experiments that taught us that excretion of antidiuretic hormone due to enlargement of the heart did not require afferent nerve signals from cardiac receptors in the control of spaceflight-induced diuresis [2]. The Stanford–NASA collaboration also provided the opportunity to conduct multiple research studies on the effect of prolonged exposure to bed rest (a ground model used to simulate the physiological effects of microgravity) on the physical work capacity of astronauts. These studies, conducted in the bed rest facility at NASA-Ames under the directorship of Dee O’Hara, were the first to involve comparisons of men and women across a large spectrum of age from 19 to 65 years [3]. The highlight of these investigations was my opportunity to be the lead exercise physiologist in the first US–Soviet collaboration bed rest study in the summer of 1979.

## University of Arizona (1982–1985)

In 1982, I followed Dr. Jack Wilmore, another of my UC Davis professors, to the Department of Exercise Science at the University of Arizona where I accepted an academic position as assistant professor and continued to focus my research efforts on physiological impacts of exercising in the heat and adaptation to the microgravity environment of space. Living in the extreme desert heat of Tucson motivated research in my interest of physiological adaptation to heat. My graduate student, Chris Kirby, conducted an intense investigation where we were able to demonstrate that heat dissipation from sweat evaporation became more efficient following acclimatization to exercise in the heat because of an increase in the ability of the sweat glands to absorb sodium [4]. We also observed this improved ability to absorb water and sodium by the kidneys; our collection of total body water intake and output along with total caloric exchange over an acclimation period of 10 days allowed us for the first time to demonstrate that the expansion of plasma volume during heat acclimatization was the result of an expansion of total body water [5] rather than the hypothesized transfer of fluid from extravascular (interstitial) space to the intravascular space [6]. Ken McKeever, one of my doctoral students who worked in the Animal Sciences department, went on to conduct experiments in dogs and horses to demonstrate that plasma volume expansion during chronic exposure to extreme heat and physical activity appeared to be a universal adaptation across species. However, physiological mechanisms appeared to be species specific, since dogs relied mainly on fluid intake through thirst [7] while horses relied primarily on improved renal tubular reabsorption of urea [8] in contrast to renal tubular sodium and water reabsorption observed in humans.

In addition to research in the area of physiological adaptation to heat, I continued my collaborative research efforts with NASA on understanding the physiology underlying the orthostatic intolerance that accompanies prolonged exposure to low-gravity environments such as those of space. With Tom Sather, another of my doctoral students, we used lower body negative pressure (LBNP) to describe the underlying physiology responsible for the ability of some individuals who tolerated orthostatic stress quite well with robust abilities to compensate for the stress of standing in gravity (we called these individuals ‘high’ tolerant) compared to others who experienced instability and fainting symptoms [9].

## NASA, Kennedy Space Center (1985–1993)

With my deep desire to translate research findings to operational application and solutions, I decided to move in 1985 from academic research to a position with NASA at the Kennedy Space Center (KSC). As the lead scientist for development of exercise countermeasures, our research focused on management of physiological deconditioning and orthostatic intolerance associated with prolonged exposure to space flight. We used the 6° head-down bed rest model to investigate mechanisms underlying the control of hemodynamics, cardiovascular function, body fluid and electrolyte shifts, renal function, metabolism, muscle, thermoregulation and the role of the autonomic nervous system in the physiological response to microgravity. Because there was no bed rest research facility located at KSC, I had the unique opportunity to collaborate with Dr. Joan Vernikos at NASA’s Ames Research Center, Dr. Robert DeBusk at Stanford University School of Medicine, and Dr. Antonio Guell at the University of Toulouse in France at the invitation of the French Space Agency, CNES, in 1990 and 1991. At least four operationally significant contributions resulted from this work. First, we demonstrated that the salt solution that astronauts were historically asked to drink before reentry in an effort to expand their blood volume was ineffective when individuals remain in microgravity [10]. Second, we showed that to maintain their level of fitness, astronauts would require more exercise during space than less fit counterparts [3]. Third, our seminal studies demonstrated that a single bout of maximal aerobic exercise could prevent for at least 24 h the orthostatic intolerance induced by 2 weeks of bed rest by increasing blood volume, restoring cardiovascular reflex function, cardiac performance and aerobic capacity [11]. This simple yet innovative approach provides a potential countermeasure for reentry and post-flight orthostatic problems that required minimal time and resources (e.g., oxygen, food, water). Finally, we were the first to obtain human muscle biopsies in a 30-day bed rest study that identified the cellular basis for reductions in fast- and slow-twitch muscle fiber size and aerobic enzymes [12]. One important finding from this study was that the tears in muscle fibers and necrotic areas seen in astronauts and believed to have been due to microgravity exposure were not seen during bed rest. This finding was later confirmed in animal flight experiments where such tears were found only after landing. These results spurned NASA to pay greater attention to the post-flight rehabilitation process.

**Fig. 3:**
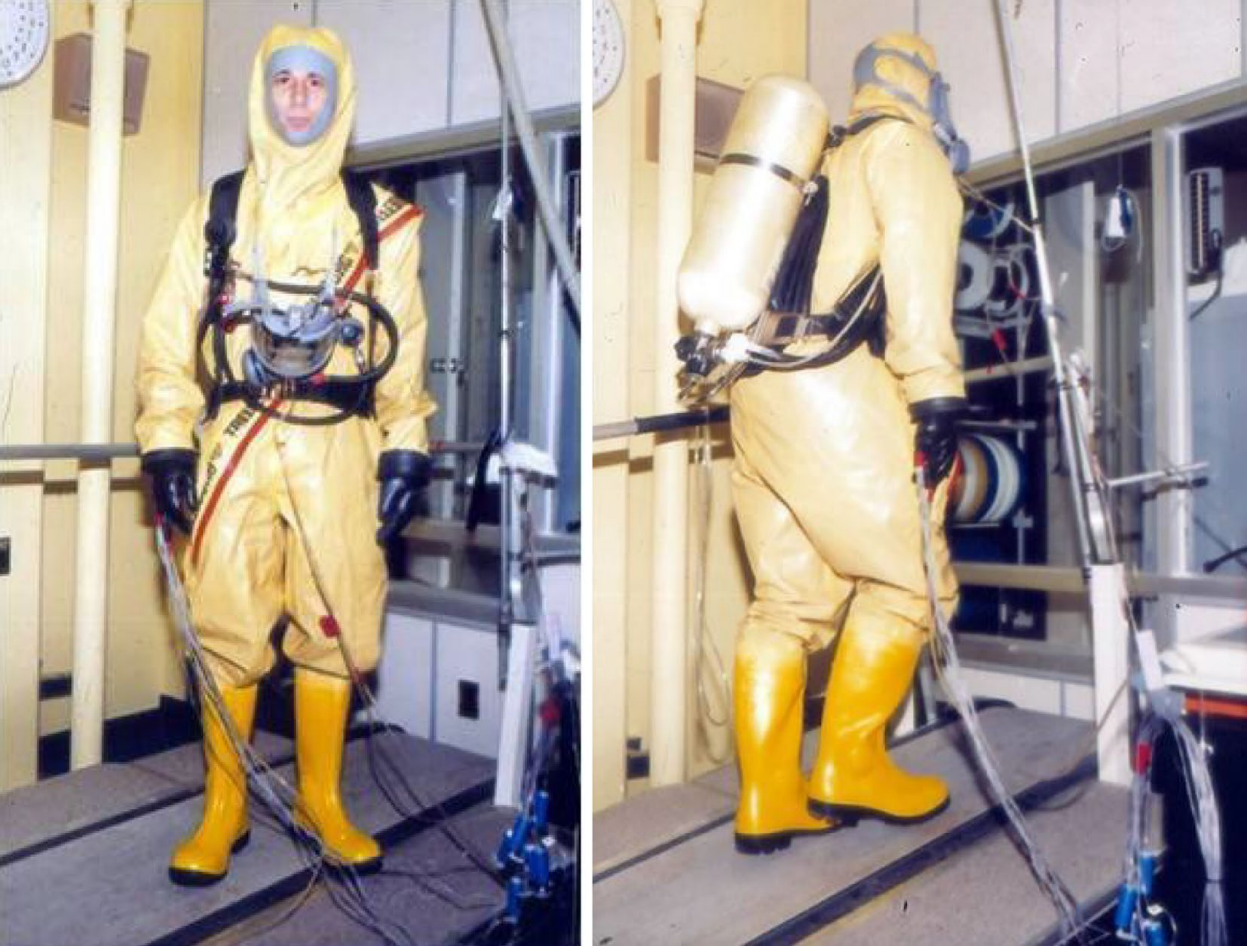
Convertino performing operational tests on an impermeable ensemble during rest (*left panel*) and physical exercise (*right panel*) conducted in the summer of 1988 in the Human Physiology Laboratory at NASA’s Kennedy Space Center

In addition to physiological research on the acute and chronic responses (adaptations) to microgravity, I had the opportunity to collaborate with Don Doerr, the former Chief of Biomedical Engineering at KSC. In this capacity, I participated in the test and development of various operational ensembles and self-contained air breathing apparatuses that enabled development of safety support and guidelines for KSC personnel (e.g., hypergolic fuel handlers, firefighters) who required working in the extreme heat and humidity created by closed impermeable suits (Fig. 3).

## US Air Force Research Laboratory (1993–1998)

With NASA’s 1992 decision to move our human research program from KSC to Johnson Space Center (Houston, Texas), I decided to accept an offer from Dr. Carter Alexander, a former NASA exercise physiologist and the Director of Plans and Programs at Brooks AFB, to take a position as a research physiologist with the US Air Force Armstrong Laboratory. In this capacity, I continued my studies on acute and chronic physiological responses to varying gravitational environments.

There was mounting evidence that the mechanisms of cardiovascular compensation during stress of gravity were more compromised in individuals with high aerobic capacity compared to their less fit counterparts [13]. Our laboratory conducted seminal studies of the influence of aerobic exercise training that provided evidence of a positive effect of endurance exercise on cardiovascular responses to gravity that reversed doctrine set by the Aerial Combat Command that high-performance aircraft pilots should not undertake endurance exercise (e.g., running) as part of their physical training regimen [14]. Our experiments also provided a physiological basis for the G-induced loss of consciousness (G-LOC) that occurs in pilots who perform the ‘check six’ maneuver (turning the head to look back over the shoulder for enemy aircraft) during aerial combat. We were the first to demonstrate in humans that side-to-side head rotation inhibits the carotid-cardiac baroreflex that plays an important role in maintaining arterial blood pressure during gravitational stress, and subsequently defends adequate cerebral perfusion [15]. In unprecedented experiments on the physiological adaptation to repeated (chronic) acceleration exposure, our experiments were the first to demonstrate that repeated high-G exposure (i.e., G training) induced cardiovascular and autonomic nervous system adaptations in humans that resulted in significant improvement in maintaining blood pressure and tolerance in subsequent high-G exposures [16]. This work provided the first physiological evidence for the phenomenon of ‘G layoff’, when G tolerance is reduced in pilots because of their ‘layoff’ from active flying. This information proved critical to the development of policy and doctrine when budget cuts forced the Air Force to consider substituting flying hours with training time in simulators. Despite anecdotal comments by pilots that their G tolerance returned to ‘normal’ following two flying exposures after G layoff, our experiments designed to measure various autonomic nervous responses demonstrated that at least three (and perhaps more) high-G training exposures are required to reach maximal G tolerance.

**Fig. 4:**
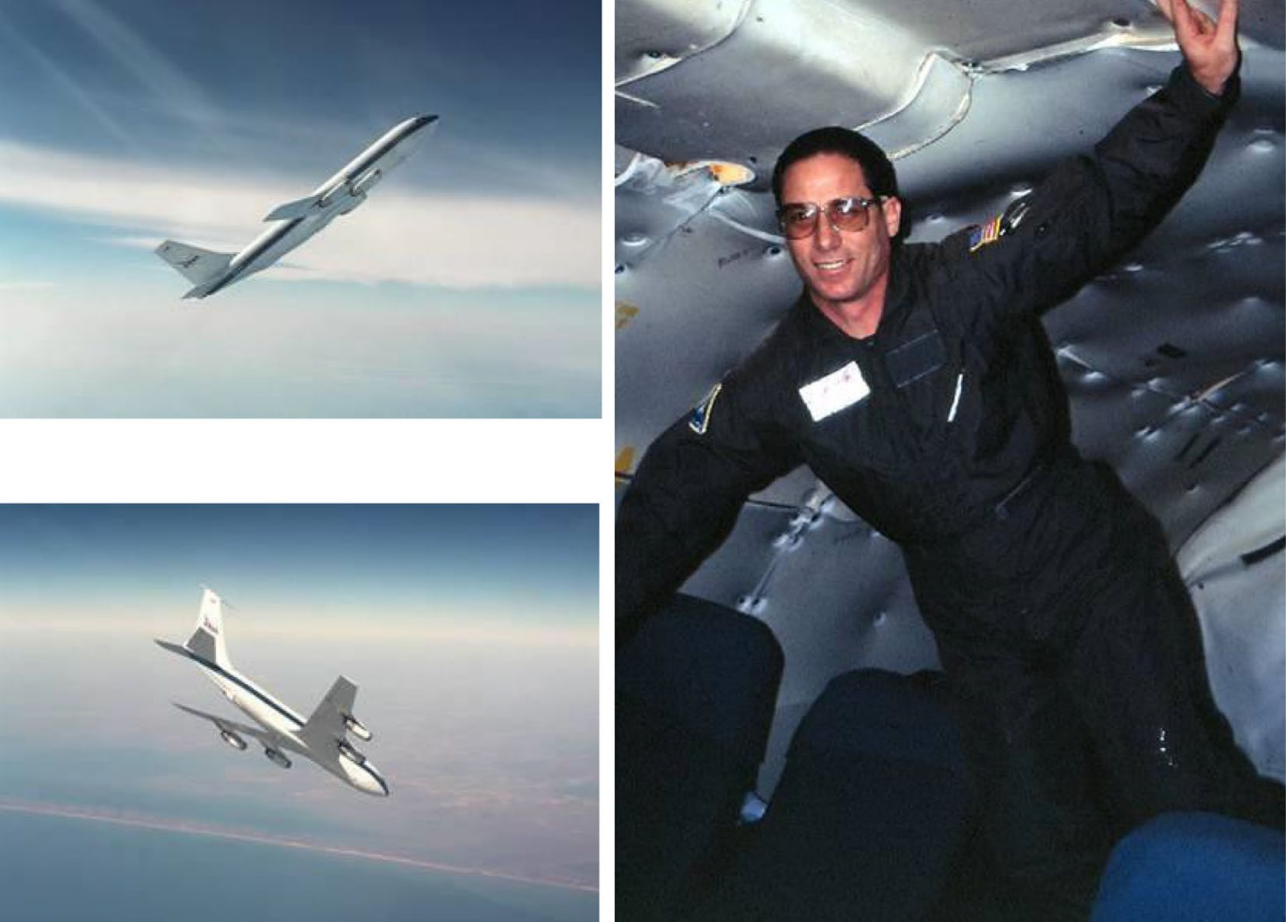
25 s of ‘weightlessness’ produced by ascent followed by rapid descent of NASA’s KC-135 aircraft (*left panels*). Convertino floating in microgravity (*free fall*) performed during January 1993 (*right panel*)

During my tenure at the Armstrong Laboratory, I had the unique opportunity to experience and study the acute effects of exposure to microgravity on cardiovascular function by participating on flight experiments under the directorship of Dr. Ricky Latham that involved hemodynamic measurements during ascents and descents on the NASA KC-135 aircraft (Fig. 4). The data collected from these experiments taken together with data obtained from humans who were exposed to acute and chronic high-G forces and prolonged bed rest provided new perspective on cardiovascular adaptation across the continuum of altered G environments [16].

## US Army Institute of Surgical Research (1998–Present)

With federal budget cuts driving decisions under the Base Realignment and Closure (BRAC) Act, our research program at Brooks AFB was terminated in 1998. With this action, I was provided an opportunity by the US Army to relocate my laboratory to their Institute of Surgical Research (USAISR) at Fort Sam Houston (San Antonio, TX). In this capacity, I continued my studies on acute and chronic physiological responses to perhaps the most extreme life-threatening environmental factor associated with reductions in central blood volume—hemorrhage induced by trauma. As such, the focus of my research on cardiovascular control during conditions of central hypovolemia was placed on advancing the emergency medical capabilities of combat medics in the pre-hospital setting of battlefield care. A primary part of this focus was to develop new clinical tools that could allow the medic to recognize that a patient was bleeding well before there was any indication from standard clinical techniques.

One of our earliest research activities was focused on providing an effective clinical tool that would control severe hemorrhage from extremity injuries. My postdoctoral fellow, Dr. Josh Wenke, conducted the first physiological testing on humans that demonstrated a deployed US Army one-handed tourniquet was not designed with the capability to stop blood flow (bleeding) in the leg [17]. The results of these tests led to the redesign of the tourniquet and the subsequent development and deployment of the Combat Applied Tourniquet (CAT) that is currently used on the battlefield and in the streets of America, and was awarded the 2005 Army’s Greatest Invention Award. The development and fielding of the CAT has and will continue to save lives of soldiers and civilians by impacting doctrine of tourniquet use.

**Fig. 5:**
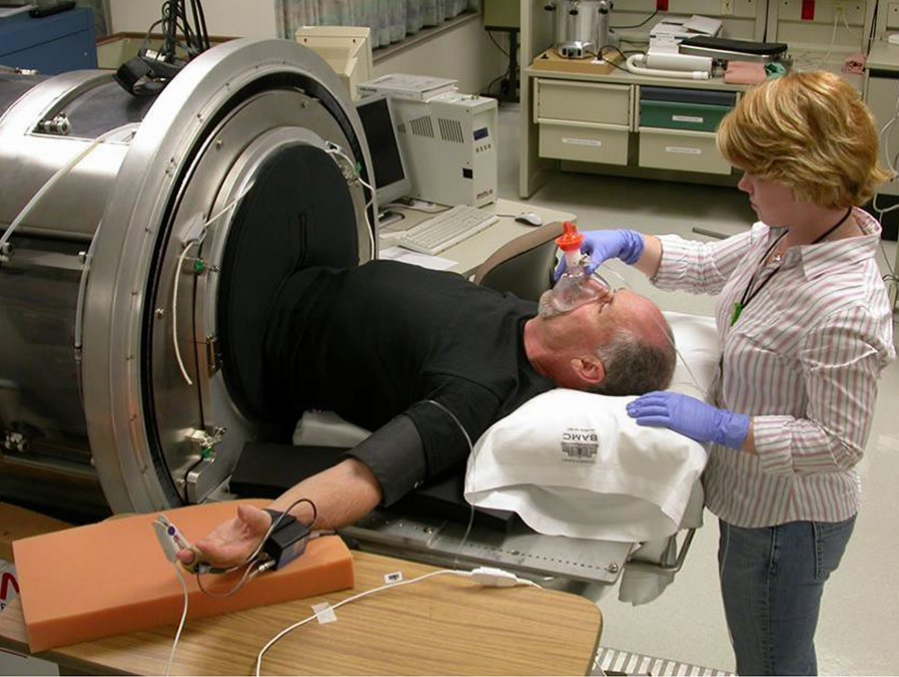
Vic Convertino undergoing a simulation of hemorrhage in the LBNP chamber during a 2006 experiment. Dr. Caroline Rickards, NRC post-doctoral fellow, is applying intrathoracic pressure regulation therapy with an impedance threshold device

In 2002, the Director of the US Army Combat Casualty Care Research Program (CCCRP) assigned me to manage a newly formed science effort that focused on pre-hospital emergency care of combat trauma casualties under fire called Tactical Combat Casualty Care (TCCC) Research. Since hemorrhage is the leading cause of death on the battlefield, our research team developed a human physiology laboratory that translated the use of LBNP into a noninvasive model for the study of human hemorrhage (Fig. 5). The capability to induce pre-syncope in all our subjects allowed for the development of a large data base (>260 humans) to model the hemodynamic, respiratory, autonomic, metabolic and coagulopathic responses during the compensatory and early decompensatory phases of hemorrhagic shock in otherwise healthy, conscious surrogates of military personnel. The findings from these studies led to new insights into our knowledge and understanding of identifying and treating progressive hemorrhage prior to the onset of overt shock, and proved critical to the conceptualization of three primary advances in extreme physiology and medicine.

**Fig. 6:**
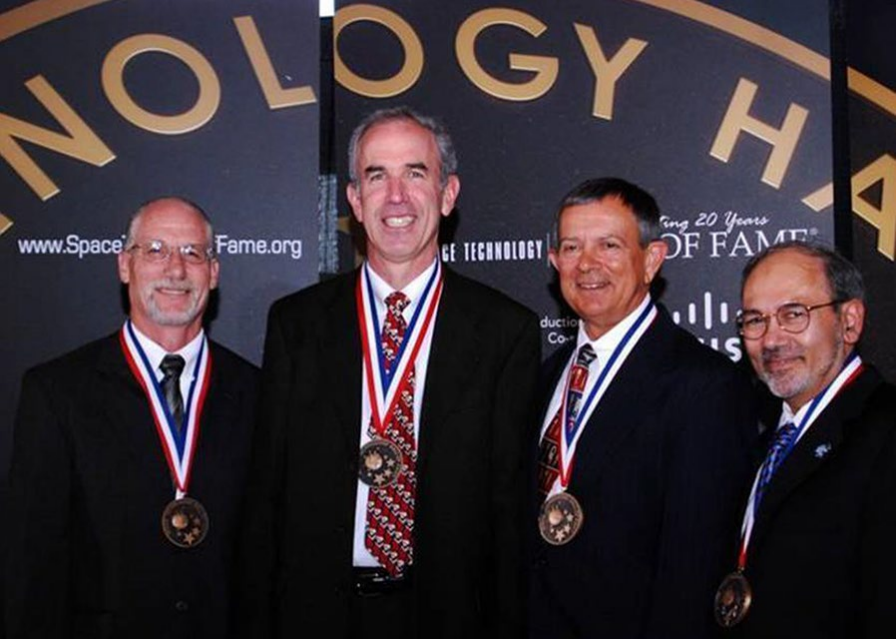
Vic Convertino, Keith Lurie, Don Doerr, and Ahamed Idris receiving their medals while attending the Space Technology Hall of Fame Induction ceremony at Colorado Springs, Colorado on April 10, 2008

First, in collaboration with Dr. Keith Lurie who developed a device that creates resistance during inspiration, we were able to find a simple operationally feasible way of counteracting syncope and hemorrhagic shock when one deals with returning astronauts or casualties on the battlefield. We demonstrated that reducing intrathoracic pressure can be an effective noninvasive resuscitation tool for restoring central blood volume as a ‘bridge’ to more definitive care where fluids may not be readily available. This therapy can be applied by having spontaneously breathing patients inspire through resistance which results in a greater vacuum within the thorax, and subsequently enhances venous return and preload to the heart in conditions of central hypovolemia (e.g., hemorrhage). In this sense, a resuscitation effect can be provided without the challenges created by carrying and infusing fluids that can dilute clotting factors or dislodge clots with elevated blood pressure. Using LBNP as our simulated progressive hemorrhage model in humans, my post-doctoral fellow Caroline Rickards demonstrated that this intrathoracic pressure regulation (IPR) therapy is capable of delaying symptoms as well as hemodynamic decompensation by protecting cerebral blood flow in addition to maintaining cardiac output [18]. Our work has led to a documented case where use of IPR therapy proved its value as a lifesaving intervention in a combat casualty. In addition to its usefulness in treating casualties with hemorrhage, the discovery that IPR therapy reduces intracranial pressure [17] speaks to its potential value as an early noninvasive intervention in the pre-hospital setting for treatment of cerebral hypoperfusion associated with traumatic brain injury. Most significantly, this technology has been placed by the US Army Tactical Combat Medical Care for use in the treatment of hypovolemic hypotension in Battalion Aid Stations and all air and land ambulances in theater, and recognized for its value to returning astronauts by its placement in the medical kit on board the Space Shuttle and International Space Station. As one of the most gratifying experiences in my career, the impedance threshold device technology used to apply IPR therapy was inducted into the Space Foundation Hall of Fame in 2008 (Fig. 6).

Second, we found that approximately one in three individuals have relatively low tolerance to reduced central blood volume [19]. These ‘low tolerant’ individuals demonstrated a physiology that was characterized by attenuated responses in tachycardia, peripheral vasoconstriction, sympathetic nerve activity, cardiac vagal withdrawal, and oscillations in blood pressure and cerebral blood flow [20, 21]. The identification of specific physiological signals that distinguish those patients at highest risk for early development of shock is one of the most fundamental observations that need to be considered in any assessment of advanced decision support and care for acute emergency settings.

Third, the recognition of individual capabilities to compensate for relative blood volume deficit during progressive hemorrhage led to the conceptualization of the ‘compensatory reserve’, a new paradigm for measuring the sum total of all compensatory mechanisms (e.g., tachycardia, vasoconstriction, breathing) that together contribute to ‘protect’ against inadequate tissue perfusion during blood loss. In our effort to discover a way to measure the compensatory reserve of individuals, our research efforts were refocused to the recognition that measurements of changes in arterial waveform features represented the integration of all cardiac and peripheral compensatory responses to hemorrhage and could provide us a tool to distinguish high- from low-tolerant individuals. By establishing collaboration with robotics engineers from the University of Colorado, our results have been translated to the development of the first prototype of a beat-to-beat ‘shock’ monitor that incorporates waveform feature extraction techniques with machine learning [22]. For the first time, tracking blood loss, early prediction of decompensatory shock, and accurate assessment of resuscitative interventions in a specific patient are possible. This technology has been shown to reduce the time required by paramedics to recognize an unstable patient by >40 % [23]. The applications of this technology to people getting out of bed after surgery, the nursing home, sports medicine or many other occupational settings are infinite. This will undoubtedly revolutionize medical monitoring, diagnosis, and interventional actions for the future of emergency medicine.

## Summary

My career in science has provided incredible opportunities to travel and meet scientists with different perspectives from around the world. Such interactions are critical to the development of a scientist, and my mentors and colleagues have been extraordinarily generous in sharing their thoughts and ideas. A cornerstone of the perspectives gained throughout my career was the experience that each success evolved out of failure(s). A second important perspective came from the approach of forming and working as part of multidisciplinary research teams with diverse talents needed to solve each problem, particularly operational problems. Physiologists, biochemists, biostatisticians, clinicians, veterinarians, engineers, electronics technicians, and computer programmers could all be found on our teams. My experience of studying the entirety of integrative systemic physiology in humans over the span of 45 years in government and academic laboratories provided me with the unique opportunity to explore how normal physiology of blood volume, circulation and blood pressure regulation in men and women of varying age and fitness levels responds and adapts to extreme conditions. I was able to take unique observations, data and novel technologies from apparently diverse sources or fields, and propose integrated hypotheses or schemes to predict how the body might react to a variety of challenges through some common pathway. Where necessary, I was able to study abnormal physiology that exists in extreme clinical conditions such as patients with heart transplants, para- and quadriplegia, chronic orthostatic hypotension, Dengue hemorrhagic fever, trauma with severe hemorrhage, or those undergoing renal dialysis or childbirth. This thorough and multifaceted approach has inevitably allowed me the most gratifying perspective of learning how to translate experimental basic physiology to operational and clinical solutions that can be applied to advance the well-being of humanity.
